# Disclosure of non-recent (historic) childhood sexual abuse: What should researchers do?

**DOI:** 10.1136/medethics-2020-106343

**Published:** 2020-11-10

**Authors:** Sergio A. Silverio, Susan Bewley, Elsa Montgomery, Chelsey Roberts, Yana Richens, Fay Maxted, Jane Sandall, Jonathan Montgomery

**Affiliations:** 1 Department of Women & Children's Health, King's College London, Westminster, London, UK; 2 Elizabeth Garrett Anderson Institute for Women's Health, University College London, Bloomsbury, London, UK; 3 Department of Midwifery, King's College London, Waterloo, London, UK; 4 Maternity Services, University College London Hospitals NHS Foundation Trust, Fitzrovia, London, UK; 5 The Survivors Trust, Rugby, Warwickshire, UK; 6 Centre for Midwifery, Child and Family Health, University of Technology Sydney, Sydney, New South Wales, Australia; 7 Faculty of Laws, University College London, Bloomsbury, London, UK

**Keywords:** ethics, women, research ethics, research on special populations, public health ethics

## Abstract

Non-recent (historic) childhood sexual abuse is an important issue to research, though often regarded as taboo and frequently met with caution, avoidance or even opposition from research ethics committees. Sensitive research, such as that which asks victim-survivors to recount experiences of abuse or harm, has the propensity to be emotionally challenging for both the participant and the researcher. However, most research suggests that any distress experienced is usually momentary and not of any clinical significance. Moreover, this type of research offers a platform for voices which have often been silenced, and many participants report the cathartic effect of recounting their experiences in a safe, non-judgemental space. With regard to the course of such research, lines of inquiry which ask adult participants to discuss their experiences of childhood sexual abuse may result in a first-time disclosure of that abuse by the victim-survivor to the researcher. Guidance about how researchers should respond to first-time disclosure is lacking. In this article, we discuss our response to one research ethics committee which had suggested that for a qualitative study for which we were seeking ethical approval (investigating experiences of pregnancy and childbirth having previously survived childhood sexual abuse), any disclosure of non-recent (historic) childhood sexual abuse which had not been previously reported would result in the researcher being obliged to report it to relevant authorities. We assess this to be inconsistent with both law and professional guidance in the United Kingdom; and provide information and recommendations for researchers and research ethics committees to consider.

## Introduction

The effects of childhood sexual abuse (CSA) are often devastating and wide ranging; affecting physical,^1^ psychological^2–4^ and sexual health.^5^ The incidence and prevalence of such abuses are difficult to ascertain, not least because most incidents of CSA go unrecognised or unreported by the victim.^6^ However, incidence rates of CSA are likely to be significant. In a representative sample from the United Kingdom (UK), 18.6% of women aged 18–24 reported contact sexual abuse (ranging from touching to rape) while they were still children.^7^ This is consistent with international data. A meta-analysis of studies from 22 countries reported some form of sexual abuse prior to the age of 18 in 19.2% of women and 7.4% of men.^8^ Where reporting does occur, numerous factors contribute to its delay or only partial disclosure.^6 9^

Researchers engage in the empirical study of survivors’ experiences in order to understand the ramifications for survivors’ social lives, their mental and physical health, and their psychosexual well-being. Findings often aim to increase public understanding of CSA,^10^ improve policies and services for survivors, and inform clinical care for people who have gone through similar experiences of CSA (whether or not this is known). The need for research is therefore clear.

Research which asks participants to discuss uncomfortable or traumatic experiences or memories is commonplace within health and psychological sciences. However, disclosure of previous trauma can occur in research environments spontaneously, even without the nature of the research being specifically focused on trauma. These disclosures can also be difficult for researchers.^11^ It is widely accepted participants may experience distress, even momentarily, when recounting their experience for research.^12^ However, evidence suggests for the large majority of participants, this distress is neither clinically significant nor longstanding.^13–17^ It has been documented that these research-based exchanges can even be cathartic due to offering a non-judgmental ‘safe space’ in which participants may achieve validation and find a voice.^18^ This has led some to conclude ‘there is a low likelihood of significant emotional harm from participating in trauma-focused studies’ for participants involved in research.^13^

A particular ethical challenge facing researchers who work with adult survivors of CSA may arise if they receive disclosures of criminal activity perpetrated on the participant which have not previously been recognised or reported to relevant authorities. This is sometimes described as non-recent (historic) CSA,^6^ even though it may not be long ago for a young adult survivor. This analysis piece was prompted by comments received (by SAS, CR, YR) from a research ethics committee (REC) on proposed work involving qualitative interviews about the childbearing experiences of women who had previously experienced CSA. The REC asked what the researchers would do if ‘specific information about criminal activities are identified *given that you would have the obligation* to report this’.^i^ The source, nature and scope of this supposed ‘obligation’ were not explained. This piece considers how researchers should respond to such a question.

## Background

The research setting of an unhurried, private interview might be the first time and place in which the participant has ever felt safe to tell their story. Most CSA is not revealed, and most adult victim-survivors of CSA choose not to make a formal report to the authorities.^19^ The reasons can be complex and numerous, ranging from rational fears of disbelief or reprisals, to wishing to protect other family members. This raises different challenges from disclosure of current abusive behaviour. Studies about experiences of non-recent (historic) CSA need an empathic researcher; research itself will be hampered if the participant fears anything they say will be reported to authorities not already privy to the information. Removing choice from any victim-survivor is deeply disempowering and re-enacts the dynamics of abuse of power and loss of control which is often characteristic of the original abuse.^4^ The process of reporting to authorities is stressful; it may result in forced disclosure to family members or partners, which the victim-survivor had not planned, which itself can be (re)traumatising. Requiring researchers to report disclosures of non-recent (historic) abuse might increase the risk of the likelihood of significant harm being caused by participation in research beyond ethically tolerable levels.

In therapeutic contexts, increasing awareness of CSA, and also of the risk of ‘gaze aversion’ (where professionals do not want to acknowledge it), has led to greater encouragement to share information with safeguarding authorities. This is conducted either via a ‘support to report’ pathway, or by breaching patient confidentiality in order to report disclosures of situations which suggest vulnerable persons are at risk.^6^ The latter route is sometimes undertaken, even where consent to report disclosures has been refused by the person disclosing their experiences of non-recent (historic) CSA.^20^ This raises significant ethical challenges.^21^ It involves a deliberate breach of the element of trust built between the patient/client (here, a victim-survivor of CSA) and the authority figure in whom they confide and who is meant to provide therapeutic counsel (whether that be a medical professional, psychological or counselling practitioner, social worker or other therapeutic practitioner).

For researchers, there is no specific guidance regarding previously unreported disclosures of non-recent (historic) CSA. Researchers must abide by their obligations to comply with general legal, ethical, and data protection standards.^6 22^ When these are ambiguous and fail to give guidance as to how choices should be made between competing principles, or on strategies to balance them fairly with each other, then researchers are ill-equipped to address the concerns raised by the REC.

## Legal positions

The suggestion which prompted this article—that if non-recent (historic) CSA which had not been previously reported was disclosed to researchers then the researcher would be obliged to report it to relevant authorities—is inconsistent with both law and professional guidance in the UK. In the UK, there is no general legal duty to report crimes, and mandatory reporting obligations are tightly defined and rare.^23^ In 2016, the government consulted on whether to make reporting of child abuse mandatory and concluded it would not do so.^24^ The Independent Inquiry into Child Sexual Abuse (IICSA) has taken evidence on mandatory reporting, but has not yet reached a conclusion on the issue.^25–27^

Existing reporting duties do not directly address the research context, but the terms on which they are formulated make it unlikely they would be interpreted such that mandatory reporting would apply. In the only clearly analogous case where reporting is mandatory in England and Wales,^28^ concerning the female genital mutilation (FGM) of minors, ‘historic’ cases are expressly excluded.^29^ In Wales, there is a wider mandatory obligation on organisations (but not individuals) to report current child protection concerns (where there is reasonable cause to suspect a child is at risk).^30^ This applies to local authorities, police, providers of probation services, local health boards, National Health Service Trusts, and Youth Offending Teams. Some researchers will be employees of such organisations, but this would be coincidental. These obligations are defined in terms of individuals being at risk, not to the investigation of crime.

Similar considerations arise even in jurisdictions which have general mandatory reporting laws, such as those that have existed in parts of Australia since 1969 and which were considered in the Royal Commission into Institutional Responses to Child Sexual Abuse.^31^ In federal Australian law, and most states, the reporting obligations are imposed only on certain authorities (as in Wales, above) and researchers are unlikely to be covered unless they work within the prescribed services. In the Northern Territories there is a more general rule that will apply to researchers as members of the public, subject to a defence of ‘reasonable excuse’ to the disclosure obligations.^32^ The arguments we make in this paper support the reasonableness of declining to disclose in order to protect the autonomy of adult research participants to determine whether reports are made. This is specifically accepted in New South Wales, also in Australia, where the law states that the fact that an adult victim does not wish the matter reported provides reasonable and sufficient justification for non-disclosure.^33^

These examples suggest that it is unlikely to be true that researchers would be legally obliged to report disclosures of historic abuse against the wishes of participants. The detailed arguments that would need to be made to support this claim will depend on the specific legal jurisdiction in which they work. We do not offer a comprehensive survey of the laws in different jurisdictions, but the illustration suffices to show that even where mandatory reporting exists, claims of an automatic or absolute legal duty to disclose are unlikely to be well founded.

## Framework for disclosure decisions in public interest

We therefore turn to consider the implications of the more general protections of privacy in both law and ethics, and in particular the way in which privacy rights are protected through the obligation of confidentiality. The starting point for analysis is that reporting of information disclosed to researchers would be unlawful and unethical, because the researcher has only acquired such information subject to legal and ethical obligations of (research) confidentiality (which adds specific responsibilities to those arising from general protections of privacy).

Any breach of said obligation would need clear justification. It is sometimes permissible, although not obligatory, to disclose information in the ‘public interest’. Any jurisdiction which recognises privacy rights will include an exception of this sort, as in the New Zealand Privacy Act 2020 which states that otherwise confidential information may be disclosed where necessary to prevent or lessen a serious threat to public health or safety; or to the life or health of individuals.^34^ In the UK there is also a recognised justification for disclosure to ‘prevent, detect, or prosecute, a serious crime’, which is generally thought to include child abuse.^35^

The implications of these powers, which are not duties such as those considered in the previous section, to breach confidentiality in the public interest can be considered against the framework set out in the General Medical Council (GMC) Guidance on Confidentiality. This was drawn up in order to ensure doctors complied with UK law, including international human rights requirements such as privacy.^36^ The GMC advises that when considering breaching confidentiality, it is necessary to balance various factors that point against disclosure, including the ‘potential harm or distress to the patient arising from the disclosure’, harm to trust in professionals and the views expressed by the patient.^36^ The GMC recognises breaching confidentiality is exceptional and should only occur if ‘failure to disclose the information would leave individuals or society exposed to a risk so serious that it outweighs the patient’s and the public interest in maintaining confidentiality’^36^ (i.e., both criteria are fulfilled). We have already seen that removing control from the participants itself causes harm and this raises the threshold required for disclosure against a patient’s objection, which the GMC suggests can only be justified in the public interest in cases where ‘failure to do so would leave others at risk of death or serious harm’.^36^ It follows that the state interest in investigating crime is insufficient to justify overriding a participant who wishes to maintain confidentiality.

The only plausible situation for a public interest disclosure in the context of research is when the participant-survivor discloses that the perpetrator of their own CSA poses a current risk to vulnerable people (especially children aged under 18). However, researchers will rarely have sufficient information to make a proper assessment of this risk and it would be unethical to proceed from suspicion to breach of confidentiality without seeking advice. This can usually be obtained on an anonymous basis (without compromising the participant’s privacy) within UK health providers via Caldicott Guardians, or through safeguarding processes. We recommend this step should precede any non-consented reporting of disclosures in order to ensure the balance is properly struck, and the researcher’s response is therefore appropriate and proportional.

## Recommendations for researchers

Researchers in the UK have no legal or ethical duty to report disclosures of previously unreported non-recent (historic) cases of CSA by their participants. We have shown why we believe that this is probably also the case for Australia where mandatory reporting laws exist. We recommend that reports should not generally be made without the consent of research participants because to take away their control over disclosure is likely to be particularly damaging in this context. Researchers should only consider reporting these disclosures if they have reason to believe the reported perpetrator of the abuse has access to, and therefore may pose a current risk to, vulnerable persons (especially children under the age of 18). This would be an example of the ‘public interest’ ground for breach of confidentiality. We recommend if researchers believe there are exceptional circumstances which make the risk to vulnerable persons so immediate, such that disclosure without consent may be necessary, the safety risk should first be assessed with an independent agency (e.g., social services or police) on an anonymous basis.

## Considerations for research ethics committees

It is important for RECs to not only assess the ethical rigour of any proposed research project, including those involving victim-survivors of CSA, but also be aware of the legal frameworks which apply to the jurisdiction in which the research is intended to be conducted. RECs should also draw researchers’ attention to the legal jurisdiction and its supposed ‘obligations’. We argue for a presumption that—in the UK at least—disclosure would be unethical whatever the legal framework. Readers or researchers working within other legal systems must carefully review the issue before planning to start. The research itself might become unethical (and re-traumatising) if the legal framework is not understood in advance, does oblige mandatory reporting, or is not made crystal clear to participants before engaging with the research. Research ethics committees should ensure researchers are able to offer all participants advice about, and appropriate signposting to, a full range of current, independent agencies and sources of support available. This can be coupled with ensuring researchers know what to do and how best to facilitate should participants wish to make contact with relevant agencies if they are not already linked into them. What we strongly oppose, however, is the enforcement of rigid protocols which encourage researchers to follow ‘support to report’ pathways, or breach participants’ confidentiality in order to report all disclosures.

Likewise, it is important for RECs to assess and ensure the suitability of the researchers undertaking sensitive projects and that of their own support networks or supervision.^11 37^ This is important especially for times when conducting a sensitive interview becomes emotionally intensive or exhaustive,^38^ challenging to conduct,^39^ or indeed, has made the researcher question whether or not there is a need to take action which would result in the breach of the participant’s confidentiality.^12^ It is therefore important for members of RECs to understand the complexity of such sensitive research, and draw on both principles of ethics and law when reaching decisions on whether or not to grant ethical approval.

## Conclusion

Childhood sexual abuse has long existed, but been taboo, and thus neglected, ignored or deemed too controversial to examine. Research must aim to understand better the experience of survivors. Those who have experienced CSA belong to a group whose voices are seldom heard, and research to better understand their experiences should be undertaken in an appropriate and sensitive manner. The analysis we present above, depicts a UK REC’s suggestion that there was an ‘obligation’ or ‘duty to report’ which was in fact misconceived in law, ethics, and policy terms. We believe the proper response to the REC’s question is that when a researcher receives a first-time disclosure of non-recent (historic) CSA, they should generally respect the participants’ decision on whether a report should be made to the police or safeguarding authorities. Only where there is clear evidence of current risk should they consider breaching confidentiality, and if they believe this to be the case, they should seek advice on an anonymous basis. They should not proceed directly to disclosure.

Our key concerns about introducing mandatory reporting are that it would undermine people’s control over the (re)telling of their stories, make them even more reluctant to participate in research, and further silence the voices of people who should be heard, without prejudice and without fear of losing control of their own story and survivorship. We have illustrated how mandatory reporting systems do recognise reasonable exceptions to the obligation to share information and why it is usually reasonable to preserve the confidentiality of disclosures by adults, of abuse they have experienced in the past. Respect for participants’ need to control whether or not data are reported to child protection authorities is an important ethical foundation enabling them to trust researchers. In the case we describe above, the UK REC to which we applied for ethical scrutiny and approvals asserted a (mythological) blanket rule which would have prevented the researchers responding sensitively to a participant who chose to disclose the experience of abuse at an ‘ethically important moment’.^40^ Such a rule would undermine, rather than promote good research ethics.

## Data Availability

There are no data associated with this work.

## Footnotes

Our emphasis.

## References

[1] Irish L , Kobayashi I , Delahanty DL . Long-term physical health consequences of childhood sexual abuse: a meta-analytic review. J Pediatr Psychol 2010;35(5):450–61. 10.1093/jpepsy/jsp118 20022919 PMC2910944

[2] Byrne J , Smart C , Watson G . "I felt like i was being abused all over again": how survivors of child sexual abuse make sense of the perinatal period through their narratives. J Child Sex Abus 2017;26(4):465–86. 10.1080/10538712.2017.1297880 28537852

[3] Oliveira AGESde , Reichenheim ME , Moraes CL , et al . Childhood sexual abuse, intimate partner violence during pregnancy, and posttraumatic stress symptoms following childbirth: a path analysis. Arch Womens Ment Health 2017;20(2):297–309. 10.1007/s00737-016-0705-6 28032212

[4] Montgomery E , Pope C , Rogers J . The re-enactment of childhood sexual abuse in maternity care: a qualitative study. BMC Pregnancy Childbirth 2015;15(1):1–7. 10.1186/s12884-015-0626-9 26306798 PMC4549944

[5] Beitchman JH , Zucker KJ , Hood JE , et al . A review of the long-term effects of child sexual abuse. Child Abuse Negl 1992;16(1):101–18. 10.1016/0145-2134(92)90011-F 1544021

[6] Rouf K , Waites B , Weatherhead S , McKail R , Manser R . Guidance document on the management of disclosures of non-recent (historic) child sexual abuse. Leicester, United Kingdom The British Psychological Society; 2016: 1–32. https://www.bps.org.uk/news-and-policy/guidance-management-disclosures-non-recent-historic-child-sexual-abuse-2016

[7] Radford L , Corral S , Bradley C , et al . The prevalence and impact of child maltreatment and other types of victimization in the UK: findings from a population survey of caregivers, children and young people and young adults. Child Abuse Negl 2013;37(10):801–13. 10.1016/j.chiabu.2013.02.004 23522961

[8] Pereda N , Guilera G , Forns M , et al . The prevalence of child sexual abuse in community and student samples: a meta-analysis. Clin Psychol Rev 2009;29(4):328–38. 10.1016/j.cpr.2009.02.007 19371992

[9] Leclerc B , Wortley R . Predictors of victim disclosure in child sexual abuse: additional evidence from a sample of incarcerated adult sex offenders. Child Abuse Negl 2015;43:104–11. 10.1016/j.chiabu.2015.03.003 25812798

[10] Mathews B , Collin-Vézina D . Child sexual abuse: raising awareness and empathy is essential to promote new public health responses. J Public Health Policy 2016;37(3):304–14. 10.1057/jphp.2016.21 27171859

[11] Dickson-Swift V , James EL , Kippen S , et al . Risk to researchers in qualitative research on sensitive topics: issues and strategies. Qual Health Res 2008;18(1):133–44. 10.1177/1049732307309007 18174541

[12] Allmark P , Boote J , Chambers E , et al . Ethical issues in the use of in-depth interviews: literature review and discussion. Res Ethics 2009;5(2):48–54. 10.1177/174701610900500203

[13] Newman E , Risch E , Kassam-Adams N . Ethical issues in trauma-related research: a review. J Empir Res Hum Res Ethics 2006;1(3):29–46. 10.1525/jer.2006.1.3.29 19385821

[14] Hewitt J . Ethical components of researcher researched relationships in qualitative interviewing. Qual Health Res 2007;17(8):1149–59. 10.1177/1049732307308305 17928485

[15] Orb A , Eisenhauer L , Wynaden D . Ethics in qualitative research. J Nurs Scholarsh 2001;33(1):93–6. 10.1111/j.1547-5069.2001.00093.x 11253591

[16] Opsal T , Wolgemuth J , Cross J , et al . "There are no known benefits ….": considering the risk/benefit ratio of qualitative research. Qual Health Res 2016;26(8):1137–50. 10.1177/1049732315580109 25857654

[17] Elmir R , Schmied V , Jackson D , et al . Interviewing people about potentially sensitive topics. Nurse Res 2011;19(1):12–16. 10.7748/nr2011.10.19.1.12.c8766 22128582

[18] McClain N , Amar AF . Female survivors of child sexual abuse: finding voice through research participation. Issues Ment Health Nurs 2013;34(7):482–7. 10.3109/01612840.2013.773110 23875549

[19] Longfield A . Protecting children from harm: a critical assessment of child sexual abuse in the family network in England and priorities for action. London, United Kingdom Children’s Commissioner; 2015: 1–109. https://www.childrenscommissioner.gov.uk/report/protecting-children-from-harm/

[20] Harrington B . Disclosure of child sexual abuse. maintaining confidentiality is not in best interest of woman or others. BMJ 1998;317(7152):208. 10.1136/bmj.317.7152.208 PMC1113554 9665919

[21] David TJ , Wynne G , Kessel AS , et al . Child sexual abuse: when a doctor's duty to report abuse conflicts with a duty of confidentiality to the victim. BMJ 1998;316(7124):55–7. 10.1136/bmj.316.7124.55 9451272 PMC2665322

[22] Richards HM , Schwartz LJ . Ethics of qualitative research: are there special issues for health services research? Fam Pract 2002;19(2):135–9. 10.1093/fampra/19.2.135 11906977

[23] Independent Inquiry into Child Sexual Abuse . Seminar briefing note. mandatory reporting: existing obligations to report child sexual abuse. London, United Kingdom Independent Inquiry into Child Sexual Abuse; 2018: 1–6. https://www.iicsa.org.uk/key-documents/7025/view/mandatory-reporting-seminar-existing-obligations-reporting-child-sexual-abuse.-briefing-note.pdf

[24] Home Office . Reporting and acting on child abuse and neglect: summary of consultation responses and government action.. London, United Kingdom HM Government; 2018: 1–35. https://assets.publishing.service.gov.uk/government/uploads/system/uploads/attachment_data/file/685463/Reporting_and_acting_on_child_abuse_and_neglect_-_response_print.pdf

[25] Independent Inquiry into Child Sexual Abuse . Mandatory reporting seminar 1 – existing obligations to report child sexual abuse: a summary report. London, United Kingdom Independent Inquiry into Child Sexual Abuse; 2018: 1–14. https://www.iicsa.org.uk/key-documents/8725/view/mandatory-reporting-seminar-one-summary-report.pdf

[26] Independent Inquiry into Child Sexual Abuse . Mandatory reporting seminar 2: a summary report. London, United Kingdom Independent Inquiry into Child Sexual Abuse; 2019: 1–17. https://www.iicsa.org.uk/key-documents/13689/view/mandatory-reporting-seminar-two-summary-report.pdf

[27] Matthews B . A model law for the mandatory reporting of child sexual abuse in England and Wales. Brisbane, Australia Queensland University of Technology; 2020: 1–15. https://www.iicsa.org.uk/document/professor-ben-mathews-model-law-mandatory-reporting-child-sexual-abuse-england-and-wales

[28] HM Goverment . Female genital mutilation act 2003, s 5B, inserted by s 71 serious crimes act 2015. London, United Kingdom Home Office; 2015: 1–129. https://www.legislation.gov.uk/ukpga/2015/9/part/5/crossheading/female-genital-mutilation/enacted

[29] Home Office, Department for Education . Mandatory reporting of female genital mutilation – procedural information (2016) para 2.1b. London, United Kingdom Home Office; 2015: 1–20. https://www.gov.uk/government/publications/mandatory-reporting-of-female-genital-mutilation-procedural-information

[30] National Assembly for Wales . Social services and well-being (Wales) act 2014, s 130. Cardiff, Wales, United Kingdom National Assembly for Wales; 2014: 1–208. https://www.legislation.gov.uk/anaw/2014/4/contents

[31] Royal Commission into Institutional Responses to Child Sexual Abuse . Final report. Barton, Australian Capital Territory, Australia Attorney-General's Department; 2017. https://www.childabuseroyalcommission.gov.au/final-report

[32] Department of the Attorney-General and Justice, Department of Territory Families, Housing and Communities . Care and Protection of Children Act 2007, Northern Territories of Australia, s. 26(3). Northern Territory, Australia Northern Territory Government; 2020: 1–166. https://legislation.nt.gov.au/en/Legislation/CARE-AND-PROTECTION-OF-CHILDREN-ACT-2007

[33] Attorney General, Minister for the Prevention of Domestic Violence . Crimes Act 1900 No. 40, New South Wales, section 316A(f). New South Wales, Australia Parliamentary Counsel's Office; 2020: 1–309. https://www.legislation.nsw.gov.au/view/html/inforce/current/act-1900-040

[34] Ministry of Justice . Privacy Act 2020, New Zealand, s 22 Principle 11(1)(f). Wellington, New Zealand New Zealand Govenment; 2020: 1–192. http://www.legislation.govt.nz/act/public/2020/0031/latest/LMS23223.html

[35] Department of Health Informatics . Confidentiality: NHS code of practice. Supplementary guidance: public interest disclosures. Leeds, United Kingdom Department of Health; 2010: 1–15. https://www.gov.uk/government/publications/confidentiality-nhs-code-of-practice-supplementary-guidance-public-interest-disclosures

[36] General Medical Council . Confidentiality: good practice in handling patient information. Manchester, United Kingdom General Medical Council; 2017: 1–78. https://www.gmc-uk.org/ethical-guidance/ethical-guidance-for-doctors/confidentiality

[37] Limerick B , Burgess‐Limerick T , Grace M . The politics of interviewing: power relations and accepting the gift. Int J Qual Stud Educ 1996;9(4):449–60. 10.1080/0951839960090406

[38] Kumar S , Cavallaro L . Researcher self-care in emotionally demanding research: a proposed conceptual framework. Qual Health Res 2018;28(4):648–58. 10.1177/1049732317746377 29224510

[39] Sands RG , Krumer-Nevo M . Interview shocks and Shockwaves. Qual Inquiry 2006;12(5):950–71. 10.1177/1077800406288623

[40] Guillemin M , Gillam L . Ethics, reflexivity, and “ethically important moments” in research. Qual Inquiry 2004;10(2):261–80. 10.1177/1077800403262360

